# Spatial distribution and determinants of household iodized salt utilization in Ethiopia: a spatial and multilevel analysis of Ethiopian demographic and Health survey

**DOI:** 10.1186/s12889-020-09538-z

**Published:** 2020-09-17

**Authors:** Yigizie Yeshaw, Adugnaw Zeleke Alem, Getayeneh Antehunegn Tesema, Achamyeleh Birhanu Teshale, Alemneh Mekuriaw Liyew, Ayenew Kassie Tesema

**Affiliations:** 1 Department of Physiology, School of Medicine, College of Medicine and Health Sciences, University of Gondar, P. O. Box 196, Gondar, Ethiopia; 2 Department of Epidemiology and Biostatistics, Institute of Public Health, College of Medicine and Health Sciences, University of Gondar, P. O. Box 196, Gondar, Ethiopia; 3 Department of Health Education and Behavioral Science, Institute of Public Health, College of Medicine and Health Sciences, University of Gondar, P. O. Box 196, Gondar, Ethiopia

**Keywords:** Iodized salt utilization, Spatial analysis, Multilevel analysis, Ethiopia

## Abstract

**Background:**

Iodine deficiency disorder is a significant public health problem, affecting both developed and developing nations worldwide. It is associated with poor body growth and irreversible mental retardation. However, little is known about the spatial distribution and determinants of household iodized salt utilization in Ethiopia. Therefore, this study aimed to explore the spatial distribution and determinants of iodized salt utilization at national level.

**Methods:**

Ethiopian Demographic and Health Survey 2016 data was used to investigate the spatial distribution and determinants of household iodized salt utilization in Ethiopia. ArcGIS 10.6 and SaTScan™ version 9.6 software were used to explore the spatial distribution and detect significant clusters, respectively. The odds ratio with its 95% confidence interval (CI) was determined for potential determinants included in the multivariable multilevel logistic regression model.

**Results:**

Household iodized salt utilization was spatially clustered in Ethiopia (Moran’s Index = 0.076, *p*-value = 0.01). The significant hotspot areas with high iodized salt utilization were located in Benishangul, Amhara, Gambella, Tigray and Northwest Oromia regions. Significant cold spot areas (areas with low iodized salt utilization) were found in Somali, and East Afar regions. Those households with higher education level ((Adjusted Odds Ratio [AOR] =1.49, 95% CI =1.14–1.93), high community level education (AOR = 1.51, 95% CI = 1.03–2.20), middle wealth index (AOR = 1.31, 95% CI = 1.04–1.65) and high community media exposure (AOR = 1.52, 95% CI = 1.07–2.17) had higher odds of iodized salt utilization.

**Conclusions:**

Household iodized salt utilization had significant spatial variation across the country**.** Both household and community level variables were found to be associated with household iodized salt utilization in Ethiopia. Therefore, increasing the education level, wealth status and community media exposure is recommended to improve iodized salt utilization in a country. A targeted intervention is also needed for those regions with low household iodized salt utilization.

## Background

Iodine is an essential micronutrient used for the synthesis of thyroid hormones, which are vital for body growth, development and control of metabolic processes in the body [1, 2]. Inadequate iodine utilization results in iodine deficiency disorder (IDD) [3], the most important preventable cause of brain damage worldwide [4–6]. The problem is pronounced for pregnant women and young children that creates a threat to the social and economic development of countries [6]. Iodine deficiency disorder can start before birth; threaten children’s mental health and their very survival. It affects body growth and mental development leading to learning disability, irreversible mental retardation, reduce school performance, poor productivity, unemployment and an increased risk of mortality [7]. Even mild iodine deficiency during pregnancy can have long-term adverse impacts on fetal cognition that are not improved by sufficient iodine intake during childhood [8–12].

Although substantial progress has been made over the last decades, worldwide, iodine deficiency remains a significant health problem; affecting both developed and developing nations [13]. Globally more than 1.88 billion people have insufficient iodine intake [14]. In Africa, over 330 million people remain at continued risk of IDD [15] and the problem is more pronounced in sub-Saharan Africa including Ethiopia [16]. In Ethiopia, the prevalence of household iodized salt utilization is 15% in 2011 [17] and 89% in 2016 [18].

Several factors are known to affect iodized salt utilization. Most importantly, being female, married [19, 20], having knowledge on IDD and iodized salt [19, 21–25], higher educational status [24, 26–28], and higher monthly income [26, 27, 29] are significantly associated with iodized salt utilization.

Iodization of salt has been proven to be the most effective strategy to provide populations with iodine and prevent IDD [30]. Hence, Ethiopia has launched mandatory salt iodization program in 2011 [31] and notable progress have made towards universal salt iodization [32]. However, still it is under the recommendation of World Health Organization (90%) [4] and its coverage and utilization varies from region to region [32, 33]. For instance, the coverage of iodized salt is lowest in Somali and Afar regions, but highest in others [18].

Despite the problem, to the best of our knowledge, there is no study that investigates the spatial distribution and determinant factors of iodized salt utilization in Ethiopia at the national level. This research is important for both policy makers and health professionals to implement targeted intervention for the problem; by identifying areas which are more affected by the problem.

## Methods

### Study area and data source

The study was conducted based on secondary data, Ethiopian Demographic and Health Survey 2016 (EDHS 2016). The EDHS is a survey conducted every five years in Ethiopia [18], a country found in East Africa (3^o^ -14^o^ N and 33^0^ – 48^o^ E), with 9 regional states (Oromia, Afar, Gambella, Benishangul-Gumuz, Amhara, Harari, Southern Nations, Nationalities and People’s (SNNP), Somali and Tigray), and two city administrations (Addis Ababa and Dire Dawa). Each region is divided into zones and the zones are again divided into Woredas. Woredas are further divided into kebeles, the smallest administrative units of the country.

The present study used the recent Ethiopian Demographic and Health Survey 2016 (EDHS 2016) aiming to determine the spatial distribution and determinants of iodized salt utilization in Ethiopia. Ethiopia Demographic Health Survey (EDHS) provides population and health indicators at the national and regional levels.

### Sample size, sampling procedure and data collection

The sampling frame used for the 2016 EDHS is the Ethiopia Population and Housing Census (PHC), which was conducted in 2007 by the Ethiopia Central Statistical Agency. The census frame is a complete list of 84,915 enumeration areas (EAs) created for the 2007 PHC. The EDHS used a two-stage stratified cluster sampling method. Stratification was done by separating the nine regional states and the two city administrations of Ethiopia, into urban and rural areas, with the exception of Addis Ababa (entirely urban). Each stratum was further divided into smaller units called enumeration areas or clusters (a geographic area consisting of 200–300 households), using the list of all clusters prepared by the 2007 Population and Housing Census (PHC) as a sampling frame. A total of 645 enumeration areas (EAs) were selected in the first stage (of which, 202 were from urban areas) using probability proportional to EA size and with independent selection in each sampling stratum. In the second stage of selection, a fixed number of 28 households per cluster were selected with an equal probability systematic selection from the newly created household listing. Data were collected by trained data collectors using a pretested structured, interviewer-administered questionnaire [18]. Households having salts in their house and tested for iodine were included. Accordingly, total weighted samples of 15,891 households were included in the study.

### Variables of the study

The outcome variable for this study was household iodized salt utilization which is dichotomized as yes (iodized) or no (not iodized). To assess the use of iodized salt, households were asked to provide a teaspoon of salt used for cooking and the salt was tested for iodine using the iodine rapid test kit [18]. Iodine rapid test kits (RTKs), can accurately distinguish between iodized and non-iodized salt and only be used to measure the percentage of salt that contains any iodine at all.

The independent variables for this study include both individual and community level factors. The individual level variables were: age of the household head, marital status, education level, wealth index, media exposure (it is a composite of reading newspaper, listening radio and watching television. By aggregating these variables we generated media exposure and recoded as yes “if a women has exposure to either of the three media sources” and no “if a women didn’t have exposure to all of the three media sources”) and residence; whereas community education level, community poverty level and community media exposure were the community level variables. The last three community-level factors were not directly found in the EDHS data; created by aggregating the selected individual level factors at the cluster level and categorized as high and low based on the median value; their value was skewed.

### Data analysis procedure

We used ArcGIS version 10.6 and Spatial Scan Statistics (SaTScanTM version 9.6) software’s to perform the spatial data analysis. Global Moran’s index (Moran’s I) was used to determine the presence of spatial autocorrelation. Hot-spot analysis was done using Getis-Ord Gi* statistics. Spatial interpolation was also done to predict the iodized salt utilization in unmeasured areas based on the values from sampled data. Spatial scan statistics was done to identify significant primary (most likely) and secondary clusters. SaTScan™ works with a moving window and requires fixing of the window size that moves across the study area. The outcome variable has Bernoulli distribution so Bernoulli model was used by applying the Kuldorff method for purely spatial analysis. Households who were utilized iodized salt were taken as cases and those who did not take were taken as controls to fit the Bernoulli model. The default maximum spatial cluster size of < 50% of the population was used as an upper limit, which allowed both small and large clusters to be detected and ignored clusters that contained more than the maximum limit. Areas with high Log Likelihood Ratio and significant *p* value were considered as areas with high iodized salt utilization compared to areas outside of the window.

To identify the determinant factors of household iodized salt utilization, STATA 14 software was used. The data were weighted before doing any statistical analysis. In EDHS, some of the regions are oversampled and some are under sampled. Therefore, we have used weighted data to restore the representativeness of the sample. Moreover, weighting for design was also done to get a reliable estimate and standard error. The whole procedure of weighting was made based on the guide of DHS statistics [34]. Since the EDHS data has hierarchical nature, measures of community variation/random-effects (Intraclass Correlation Coefficient, Median Odds Ratio and Proportional Change in Variance) were estimated. The values of these measures were significant, indicating the use of multilevel logistic regression model than ordinary logistic regression. Model comparison was done using deviance between the null-model (a model with no independent variable), model I (a model with only individual-level factors), model II (a model with community-level factors) and model III (a model that contain both individual and community level independent variables). A model with the lowest Deviance (model III) was the best fitted model. Both bivariable and multivariable multilevel logistic regression was performed to identify the determinant factors of household iodized salt utilization in Ethiopia. All variables with a p value < 0.25 at bi-variable multilevel logistic model analysis were entered into the multivariable multilevel logistic regression model. *P* value ≤0.05 was used to declare statistically significant variables in the final model.

## Results

### Sociodemographic characteristics

Weighted sample of 15,891 households were included in this study. The majority, 12,731(80%) of the households were rural. The mean age of household head was 44.38 years (± 16.10 SD). More than half, 8703(54.77%) of the households had no education. The majority (71.53%) of the households had no radio. Regarding marital status, more than three fourth (77.33%) of the households were currently married. Eight thousand three hundred and four (52.25%) of them were from low community education. Eight thousand three hundred and eleven (52.30%) of them were from community with high poverty level (Table 1).

**Table 1:** Sociodemographic characteristics of respondents in Ethiopia, 2016

Variables	Weighted frequency	Percent
Residence		
Urban	3160	20.00
Rural	12,731	80.00
Age (years)		
< 25	905	5.69
25–34	3957	24.90
35–44	3747	23.58
45–54	2594	16.33
55–64	2352	14.80
65 and above	2336	14.70
Sex of household head		
Male	11,821	74.39
Female	4070	25.61
Marital status		
Never married	649	4.08
Currently married	12,288	77.33
Formerly married	2954	18.59
Educational status		
No education	8703	54.77
Primary education	4849	30.52
Secondary education	1250	7.87
Higher education	1089	6.85
Wealth index		
Poorest	3001	18.89
Poorer	3067	19.30
Middle	3032	19.08
Richer	2992	18.83
Richest	3799	23.91
Media exposure		
Yes	4525	28.47
No	11,366	71.53
Community education level		
Low	8304	52.25
High	7587	47.75
Community poverty level		
Low	7580	47.70
High	8311	52.30
Community media exposure level		
Low	8151	51.29
High	7740	48.71

### Spatial analysis of household iodized salt utilization

**Fig. 1:**
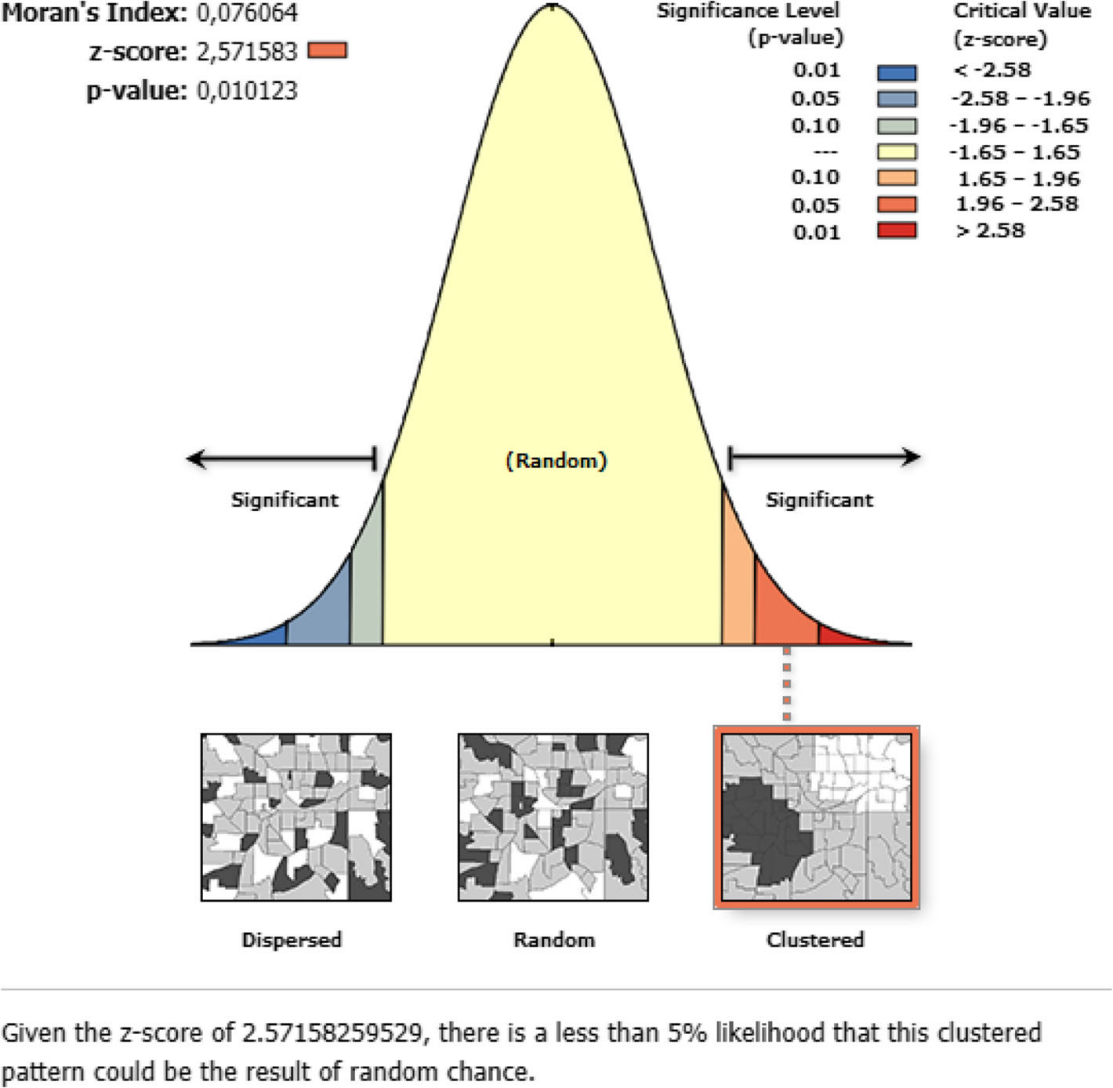
Global spatial autocorrelation of household iodized salt utilization in Ethiopia, 2016 (Using Arc-GIS version 10.6 and SaTScan version 9.6 software)

**Fig. 2:**
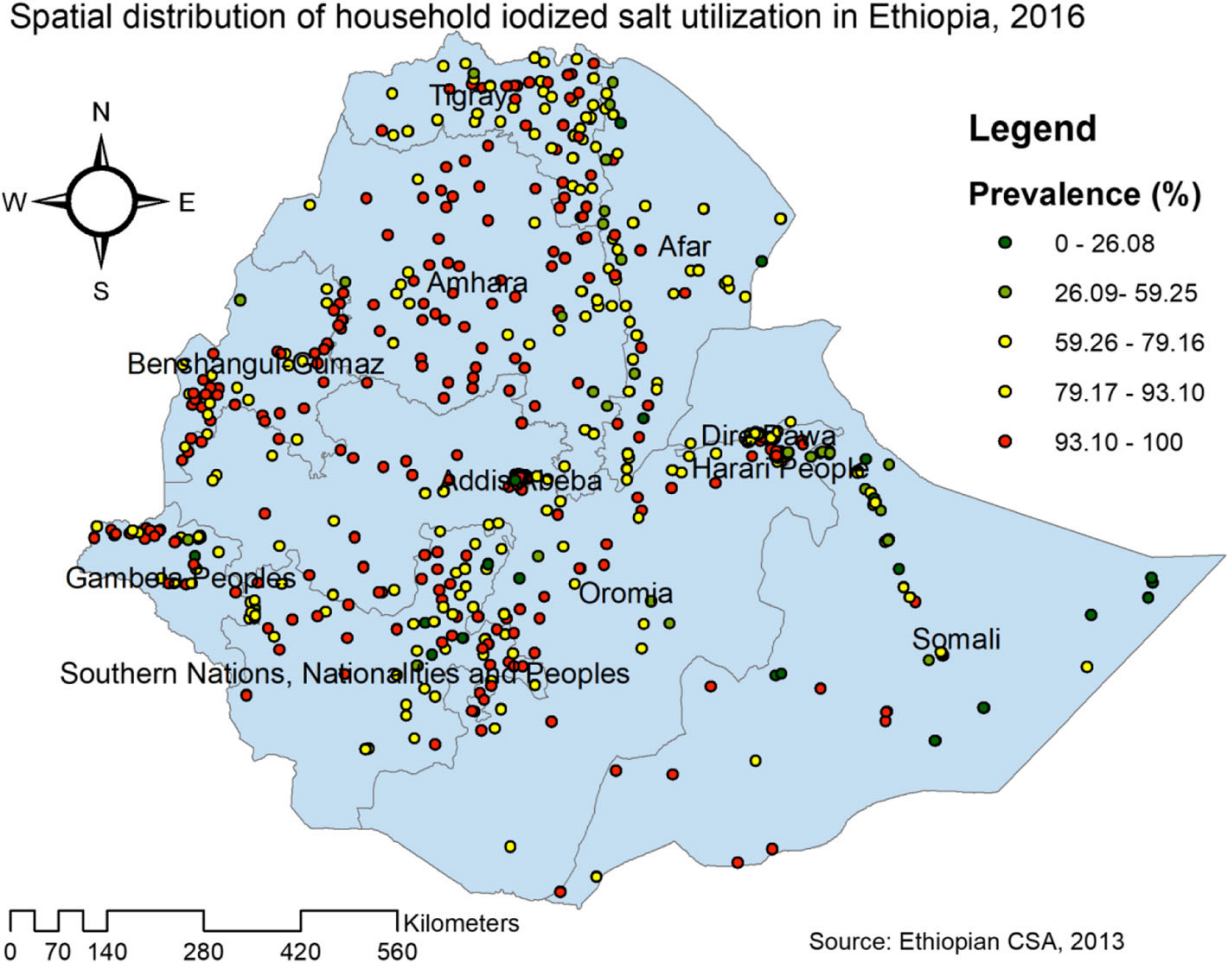
Spatial distribution of household iodized salt utilization across regions in Ethiopia, 2016 (Using Arc-GIS version 10.6 and SaTScan version 9.6 software)

The overall iodized salt utilization in Ethiopia was 89.27% (95% CI = 88.78–89.74%). Among regions, Benishangul had the highest prevalence of household iodized salt utilization (94.14%); whereas the lowest was in Somali region (62.45%). The spatial distribution of household iodized salt utilization in Ethiopia was non-random (Global Moran’s I = 0.076, p value = 0.01) (Fig. 1). The highest prevalence of household iodized salt utilization was identified in the Amhara, Tigray, Benishangul Gumuz, Gambela, Oromia and central SNNPRs regions whereas low iodized salt utilization was located in the entire Afar and Somali regions (Fig. 2).

**Fig. 3:**
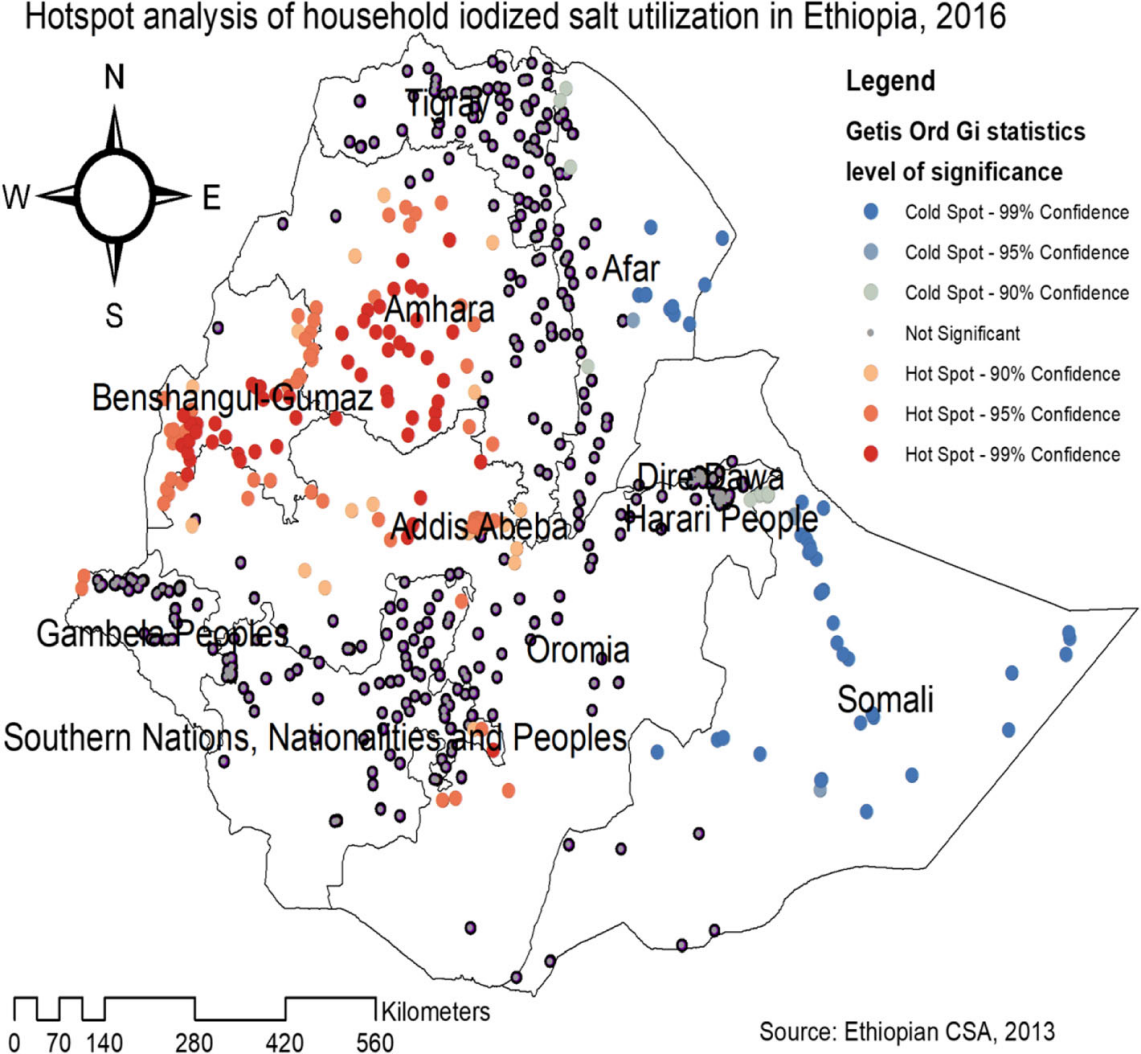
Getis Ord Gi statistical analysis of hotspot areas of household iodized salt utilization in Ethiopia, 2016 (Using Arc-GIS version 10.6 and SaTScan version 9.6 software)

**Fig. 4:**
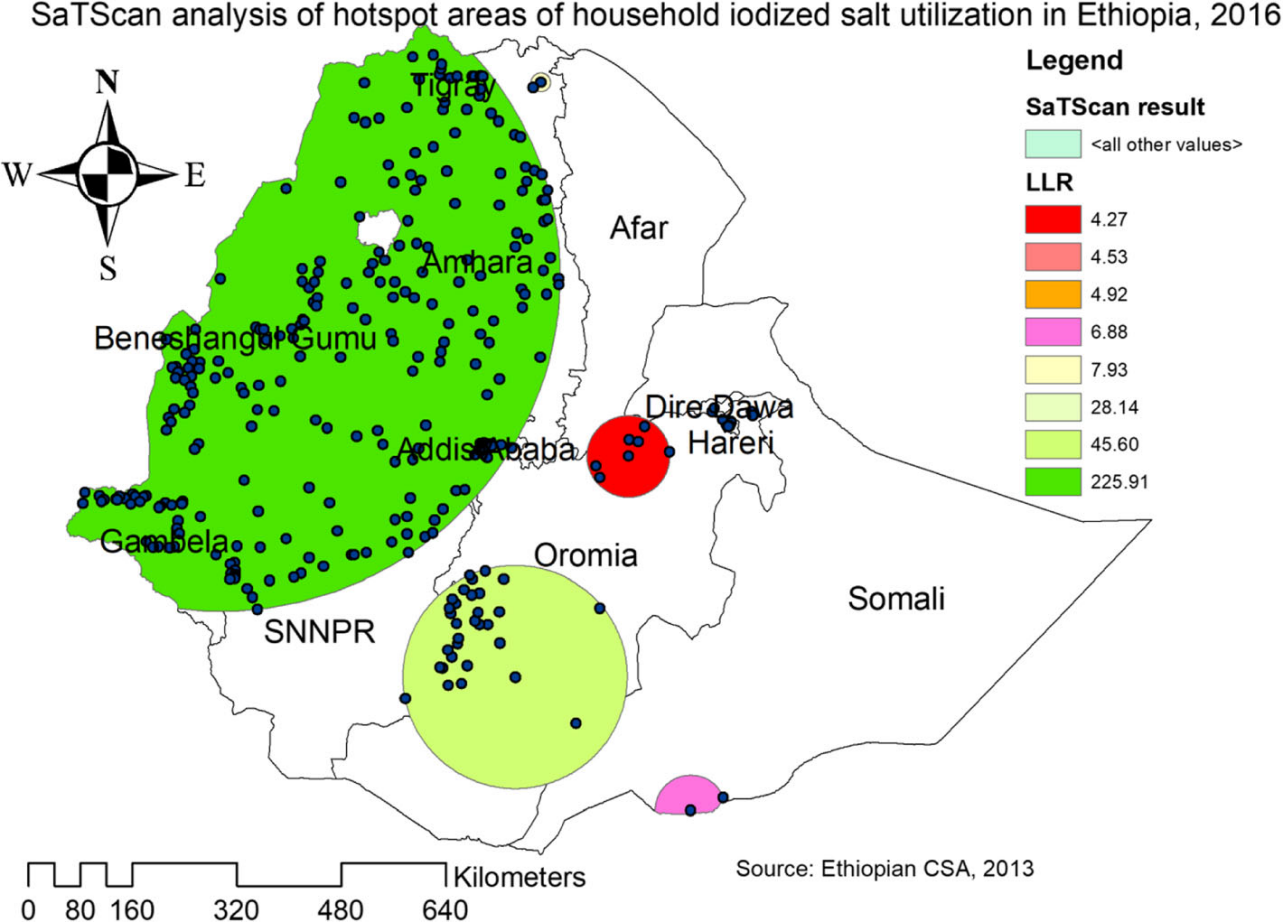
SaTScan analysis of primary and secondary hotspot clusters of household iodized salt utilization in Ethiopia 2016 (Using Arc-GIS version 10.6 and SaTScan version 9.6 software)

The Getis Ord Gi statistical analysis shows the hotspot and cold spot areas of household iodized salt utilization in Ethiopia. The red colors indicates the significant hotspot areas (higher rates of household iodized salt utilization), which was found in the central and southern parts of Amhara, Northeast part of Benishangul-Gumuz, Addis Ababa, Southwest Oromia and West Gambella regions. In contrast, the blue color indicates significant cold spot areas (areas with low iodized salt utilization), found in Somali, and East Afar regions (Fig. 3). In the SaTScan analysis, a total of 364 significant clusters were identified. Of these, 304 were primary clusters. The primary clusters were located in the entire Benishangul, Amhara, Gambella, Tigray and Northwest Oromia regions, centered at 11.340042 N, 35.126734 E with 509.53 km radius, a Relative Risk (RR) of 1.15, and Log-Likelihood Ratio (LRR) of 225.9, at *p*-value< 0.001. Households within the spatial window had 1.15 times higher odds of utilizing iodized salt compared to those households outside the spatial window (Table 2). The secondary clusters were located in Southwest Oromia, eastern SNNPR, Dire Dawa, Harari, and Southwest Somali regions (Fig. 4).

**Table 2:** Significant SaTScan clusters of areas with high proportion of household iodized salt use in Ethiopia, 2016

clusters	Enumeration areas (EAs)/ clusters detected	Coordinates/radius	Population	Cases	RR	LLR	P-value
1 (number of clusters)	256, 457, 35, 364, 137, 324, 244, 386, 415, 285, 183, 569, 409, 209, 36, 548, 407, 533, 559, 541, 563, 246, 150,615, 65, 595, 602, 515, 498, 581, 259, 335, 508, 433, 203, 317, 6, 165, 184, 52, 124, 416, 88, 621, 17, 320, 361, 374, 109, 349, 395, 462, 494, 70, 516, 3, 275, 292, 431, 304, 193, 279, 382, 169, 643, 73, 175, 161, 429, 167, 248,375, 158, 474, 403, 294, 24, 163, 640, 512, 456, 638, 558, 132, 280,504, 531, 120, 327, 218, 296, 312, 399,152, 612, 63, 411, 549, 47, 291, 469, 114, 221, 322, 482, 229, 206, 231, 315,234, 350, 346, 105, 106, 69, 38, 426, 555, 343, 567, 603, 448, 104, 260, 592,253, 199, 627, 233, 370, 507, 265, 628, 80, 309, 62, 258, 517, 536, 176, 118, 435, 266, 593, 545, 219, 485, 10, 23, 618, 510, 586, 119, 460, 425,267, 177, 446, 13, 270, 188, 284, 326, 417, 583, 432, 340, 66, 554, 423, 46, 262, 268, 591, 572, 489, 299, 542, 551, 401, 354, 98, 526,142, 243, 617, 459, 552, 616, 465, 168, 255, 181, 371, 197, 174, 478, 528, 274, 78, 486, 154, 76, 11, 339, 463, 147, 477, 107, 145, 91, 31, 532, 100, 447, 369, 626, 584, 487, 608,112, 314, 108, 144, 645, 207, 59, 195, 300, 635, 170, 305, 153, 464, 414,582, 15, 159, 110, 227, 247, 639, 302, 225,293, 579, 156, 19, 61, 264, 261, 437, 475, 428, 155, 451, 509, 539, 287, 560,330, 502, 575, 211, 139, 90, 577, 402, 252, 136, 236, 410, 636, 83, 597, 353, 392, 204, 400, 217, 325, 18, 359, 538, 200, 303, 310, 496, 590, 97, 424, 143, 455, 81, 351, 223, 271, 449, 611, 331, 345, 442, 40, 376	(11.340042 N, 35.126734 E) / 509.53 km	7522	6923	1.15	225.9	*p* < 0.001
2	422, 34, 316, 398, 405, 468, 600, 232, 21, 518, 445, 313, 32, 182, 576, 574, 377, 634, 215, 365, 12, 216, 408, 26, 148, 308, 391, 289, 589, 578, 50	(5.844300 N, 39.182881 E) / 170.92 km	790	759	1.12	45.6	p < 0.001
3	610, 383, 329, 238, 495, 381, 443, 173, 396, 60, 393, 288, 614, 28, 228, 157, 397, 56, 387, 257, 419, 357, 534, 44, 473, 179, 58, 418, 29	(9.370004 N, 42.102751 E) / 7.94 km	698	661	1.1	28.1	p < 0.001
4	341, 226	(14.041828 N, 39.537923 E) / 14.42 km	53	53	1.16	7.9	*P* = 0.166
5	208, 520	(4.006703 N, 41.599741 E) / 53.81 km	46	46	1.16	6.1	*P* = 0.397
6	1, 566	(9.505470 N, 42.438628 E) / 5.85 km	54	53	1.14	4.9	*P* = 0.893
7	166, 311	(9.510583 N, 41.898978 E) / 5.60 km	51	50	1.14	4.5	*P* = 0.966
8	476, 506, 412, 122, 333, 245, 372	(8.888553 N, 40.744565 E) / 62.70 km	174	162	1.08	4.3	*P* = 0.983

**Fig. 5:**
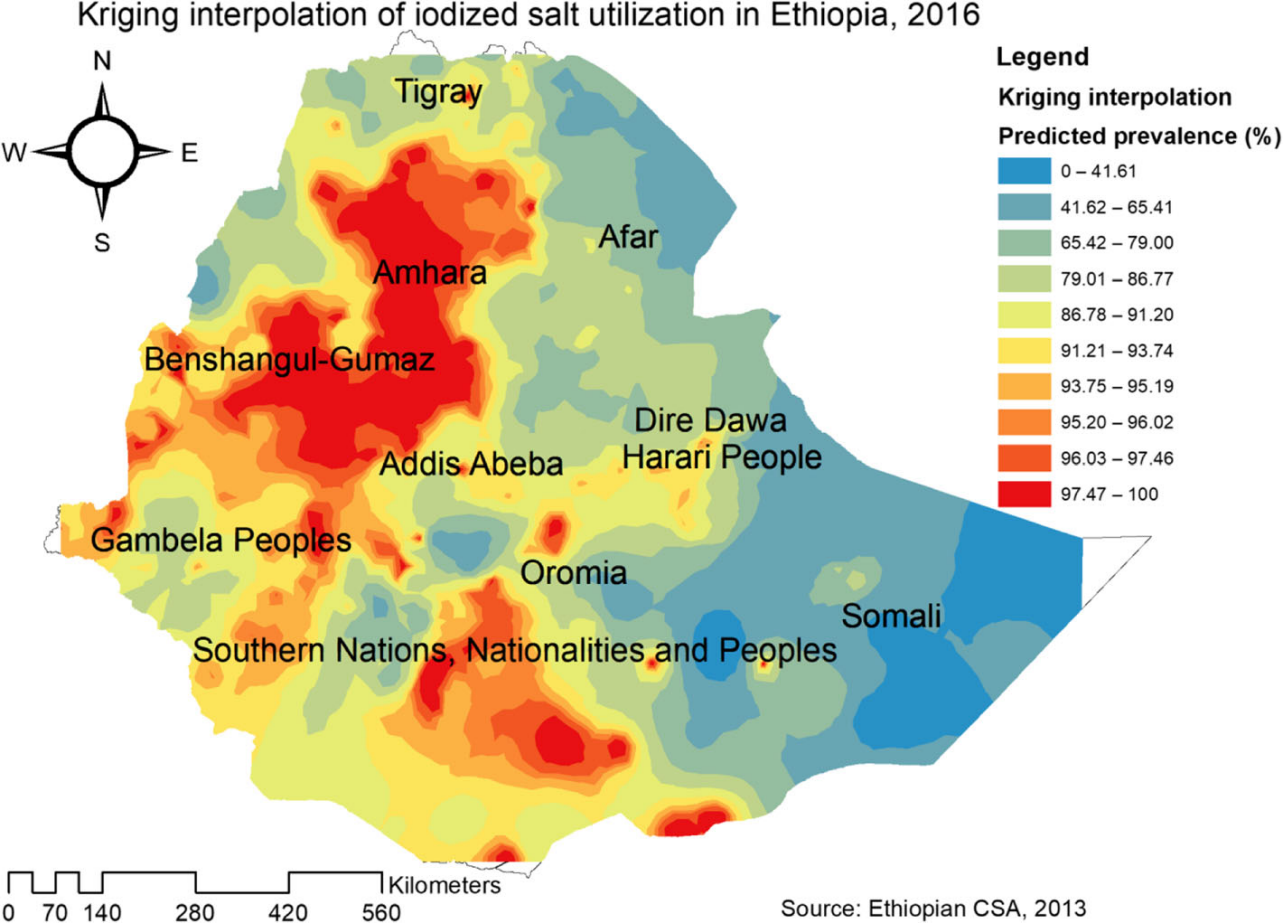
Kriging Interpolation of household iodized salt utilization in Ethiopia, 2016 (Using Arc-GIS version 10.6 and SaTScan version 9.6 software)

In the Kriging interpolation, central, eastern and Southern parts of Amhara, north and southwest parts of Oromia and eastern part of Benishangul have predicted areas of high household iodized salt utilization compared to other regions. In contrast, predicted lower household iodized salt utilization areas were found in eastern Afar and Somali regions (Fig. 5).

### Random effect analysis

The results of the random-effects model indicated that there was significant clustering of household iodized salt utilization across the communities (OR of community level variance =3.442, 95% CI = 2.905–4.078). The intra-class correlation (ICC) in the null model indicated that 51.1% of the overall variability of household iodized salt utilization was attributed to cluster variability. The median odds ratio (MOR) for household iodized salt utilization was 5.82 in the null model, which indicates that there was a variation in iodized salt utilization between clusters. This means if we randomly select households from different clusters, households at the cluster with higher household iodized salt utilization had 5.82 times higher odds of iodized salt use as compared with those households at cluster with lower iodized salt use. The Proportional Change in Variance (PCV) also increases from 6.3% from model I to 9.4% in model III (a model with individual and community level variables), which indicates the last model (model III) best explains the variability of household iodized salt utilization, Besides, model fitness was checked using deviance and the model with the lowest deviance (model III) was the best fitted model (Table 3). Moreover, the predictive ability of these models was checked using Receiver Operating Curve (AUC), plotted based on sensitivity and 1 – specificity. Accordingly, the Area Under the ROC curve for model III was the highest (62.3%), Therefore, incorporating the community level variables has improved the model.

**Table 3:** Model comparison and random effect analysis result

Parameters	Null model	Model I	Model II	Model III
Community-level variance	3.442	3.224	3.173	3.120
ICC	51.1%	49.5%	49.1%	48.7%
MOR	5.82	5.53	5.43	5.36
PCV	Ref	6.3%	7.8%	9.4%
Log likelihood	− 5023.507	− 5003.621	− 5006.784	− 4995.235
LR test	X^2^ = 2629.15 P < 0.001	X^2^ = 2389.61 P < 0.001	X^2^ = 2419.65 P < 0.001	X^2^ = 2334.39 P < 0.001
Deviance	10,047.01	10,007.242	10,013.568	9990.470

### Determinants of household iodized salt utilization in Ethiopia

On bivariable multilevel logistic regression analysis, education level, wealth index, media exposure, residence, community education level, community poverty level, and community media exposure level were associated with household iodized salt utilization (*p* < 0.25). Therefore, these variables were eligible for the multivariable multilevel logistic regression/final model. Accordingly, wealth index, education level, community media exposure level and community education level were significantly associated with household iodized salt utilization in Ethiopia (*p* ≤ 0.05).

The odds of iodized salt utilization among the households with higher education level were 1.5 times higher compared with those who had no education (AOR = 1.49, 95% CI =1.14–1.93). Those households who were from high community education level had 1.5 times higher chance of iodized salt utilization compared with households of low community education level (AOR = 1.51, 95% CI = 1.03–2.20). The odds of household iodized salt utilization was 1.3 times higher among households with middle wealth index compared with the poorest once (AOR = 1.31, 95% CI = 1.04–1.65). The odds of iodized salt utilization among households with high community media exposure level was 1.5 times higher compared with households from low community media exposure level (AOR = 1.52, 95% CI = 1.07–2.17) (Table 4).

**Table 4:** Multilevel logistic regression analysis of household iodized salt utilization in Ethiopia, 2016

Variables	Use of iodized salt		Odds ratio	
	Yes N (%)	No N (%)	COR (95% CI)	AOR (95% CI)
Community media exposure level				
low	7127(50.24)	1024(60.08)	1	1
High	7059 (49.76)	681 (39.92)	2.14 (1.57–2.91)	1.52 (1.07–2.17)*
Residence				
Rural	11,284(88.63)	1447 (11.37)	1	1
Urban	2902(91.84)	258 (8.16)	1.81 (1.29–2.55)	0.82 (0.50–1.35)
Media exposure				
Yes	4120 (91.06)	405(8.94)	1.21 (1.10–1.38)	1.10 (0.95–1.26)
No	10,066(88.56)	1300 (11.44)	1	1
Education level				
No education	7734 (88.87)	969 (11.13)	1	1
1^ry^ education	4312 (88.92)	537 (11.08)	1.10 (0.95–1.24)	1.02 (0.89–1.17)
2^ry^ education	1119 (89.56)	131 (10.44)	1.39 (1.12–1.74)	1.25 (0.99–1.57)
Higher education	1021 (93.73)	68 (6.27)	1.68 (1.30–2.16)	1.49 (1.14–1.93)*
Community education				
Low	7419 (89.35)	885(10.65)	1	1
High	6767(89.19)	820 (10.81)	2.16 (1.59–2.94)	1.51 (1.03–2.20)*
Community poverty				
High	7455 (89.71)	855 (10.29)	1.99 (1.46–2.71)	1.20 (0.78–1.84)
Low	6731 (88.79)	850 (11.21)	1	1
Wealth index				
Poorest	2577 (85.86)	424 (14.14)	1	1
Poorer	2762 (90.07)	305 (9.93)	1.22 (0.99–1.49)	1.15 (0.94–1.41)
Middle	2711 (89.40)	321 (10.60)	1.45 (1.16–1.81)	1.31 (1.04–1.65)*
Richer	2672 (89.29)	320 (10.71)	1.49 (1.18–1.88)	1.26 (0.99–1.62)
Richest	3465 (91.21)	334 (8.79)	1.79 (1.39–2.29)	1.26 (0.92–1.73)

*p ≤ 0.05

## Discussion

This study aimed to explore the spatial distribution and determinant factors of household iodized salt utilization in Ethiopia. Accordingly, we found that household iodized salt utilization in Ethiopia was clustered and affected by different factors.

The spatial distribution of household iodized salt utilization in Ethiopia was none random. The significant hotspot areas with high iodized salt utilization were located in entire Benishangul, Amhara, Gambella, Tigray and Northwest Oromia regions. In contrast, lower household iodized salt utilization was observed in Somali and Afar regions indicating the need to increase iodized salt coverage and so its utilization with strong collaboration between the governments, salt processing industries, supporting organizations and the community. The possible reason for this observed non-random distribution of iodized salt in the country might be due to the differences in access to non-iodized salt sources (high in Afar and Somali regions) [35], post fortification loss, and distance from fortification site. Another probable justification for this non-random distribution of iodized salt utilization could be the difference in education level of the households. In this study, only 106 (22.94%) of Somali, and 37 (27.94%) of Afar households have primary and above education, which is lower than other regional states of Ethiopia. This low educational status of the two regions might lead to low prevalence of household iodized salt utilization in these regions. This is because low education level is usually associated with low level of community awareness on prevention of IDDs, the benefits of iodized salt and its utilization [26, 27].

In this study, wealth index, education level, community education level and community media exposure level were significantly associated with higher odds of household iodized salt utilization in Ethiopia (p ≤ 0.05). The odds of iodized salt utilization among households with higher education level were 1.5 times higher compared with those who had no education. Similarly, those households who were from high community education level had higher chance of iodized salt use compared with households from low community education level. This finding is similar with the finding of other studies in Ethiopia [24, 26–28]. This could be due to participants who had education are more likely to be knowledgeable on the use of iodized salt than those who did not have education [26, 36–38]. Moreover, households who had education usually had employment opportunities that might lead to relatively better socioeconomic status to purchase and use iodized salt compared to those households with no education.

Respondents’ wealth index status is also associated with household iodized salt utilization in Ethiopia. The odds of iodized salt utilization among households with middle wealth index were higher compared with the poorest once. Our finding is consistent with the finding of many other studies in Ethiopia [19, 26, 27, 29]. This might be due to the reason that those households with poorest wealth index are facing economic constraint for buying iodized salt; iodized salt is less likely to be found in poor households due to its relatively higher cost [39].

Another factor associated with households iodized salt utilization in this study is level of community media exposure. The odds of iodized salt utilization among households with high community media exposure level were 52% times higher compared with households from low community media exposure level. The current finding is in line with a study in Turkey [37]. This is due to those households who had media exposure usually have more information about their health including the importance of iodized salt compared to households without media exposure. This finding implies that health education through mass media such as radio has been effective in promoting the use of iodized salt especially in rural communities [40]. This would be more effective for countries like Ethiopia, a country where more than 80% of its population is rural in resident and had no access to use latest mass media types.

This study has the following strengths. Firstly, we used multilevel model, a model that accounts the hierarchical nature of the data to get reliable estimates. The second strength of this study is the use of nationally representative data which can have greater power of generalizability. As limitation, this study uses rapid test kits (RTKs) essential only to determine the presence or absence of iodine, are non-quantitative. Therefore, we are unable to determine the quality of salt iodization and its spatial distribution in the country.

## Conclusions

Household iodized salt utilization had significant spatial variation across the country. Benishangul-Gumz, Amhara, Gambella, Tigray and Northwest Oromia regions were the significant hotspot areas with high iodized salt utilization. Households with higher education level, from high community education level and high community media exposure level and middle wealth index had higher odds of iodized salt utilization. Therefore**,** increasing education level, wealth status and community media exposure is recommended to improve iodized salt utilization. Moreover, targeted intervention is needed for Afar and Somali regions, areas with low iodized salt utilization.

## Data Availability

All relevant data are included in the article. The Ethiopian Demographic and Health Survey data set used for the analysis was obtained from the link https://dhsprogram.com/data/dataset_admin/index.cfm, after reasonable request of the DHS Program.

## Abbreviations

AOR: Adjusted Odds Ratio
CI: Confidence Interval
COR: Crude odds ratio
EDHS: Ethiopian Demographic and Health Survey
ICC: Intraclass Correlation Coefficient
IDD: Iodine Deficiency Disorder
MOR: Median Odds Ratio
PCV: Proportional Change in Variance
